# Salvage Therapy in Early Relapse of T-Lymphoblastic Leukemia/Lymphoma Using Daratumumab/Nelarabine Combination: Two Consecutive Cases

**DOI:** 10.1155/2022/9722787

**Published:** 2022-01-10

**Authors:** Ingolf Molle, Irma Petruskevicius, Peter Kamper, Francesco d'Amore

**Affiliations:** Department of Haematology, University Hospital of Aarhus, Aarhus, Denmark

## Abstract

Treatment of early relapses of T lymphoblastic leukemia/lymphoma is often unsuccessful. We tested an experimental regimen containing daratumumab and nelarabine in two young patients with early relapses of T lymphoblastic lymphoma and T-ALL, respectively. Both patients achieved a deep complete remission. Combining daratumumab with chemotherapy may have a role in relapsing T lymphoblastic leukemia/lymphoma.

## 1. Introduction

Relapsing T lymphoblastic lymphoma/leukemia has a poor prognosis with up to 10% survivors after 2 years [1, 2]. In early relapses, the prognosis is extremely poor [1].

In relapsing young patients, possible remission induction treatments include traditional high-dose chemotherapy, such as FLAG-IDA or combination chemotherapy based on high-risk ALL protocols. Nelarabine, the pro-drug of the deoxyguanosine analogue 9-beta-d-arabinofuranosylguanine used alone or more recently in combination chemotherapy, is another option [3].

Results are far from satisfying with roughly one-third of patients achieving a new remission [1, 2]. Second remissions are often short-lived, and subsequent relapses hamper stem cell transplant (SCT) in many patients.

T lymphoblasts usually express high levels of CD38 on the cell surface [4]. The human IgG1k monoclonal anti-CD38 antibody daratumumab has proven very potent in multiple myeloma, and a few observations have suggested a possible future role in T lymphoblastic leukemia/lymphoma [5–9].

We tested an experimental regimen based on daratumumab/nelarabine combination in two early relapses of T lymphoblastic leukemia/lymphoma. Both cases strongly expressed CD38 on the majority of the cancer cells, but a fraction of blasts was CD38 negative. A combination of daratumumab and nelarabine was therefore chosen.

## 2. Case 1

A 38-year-old male with no medical history presented with T lymphoblastic lymphoma with a large mediastinal bulk tumor but no bone marrow or CNS involvement. Though not registered in the NOPHO database (no bone marrow involvement), he was treated with NOPHO2008 high-risk induction (dexamethasone, daunorubicin, and vincristine) and achieved a complete structural and metabolic response after 30 days. He received consolidation chemotherapy per protocol (NOPHO.org), but unfortunately he had an early relapse during high-risk maintenance after 1 year of intense poly-chemotherapy. Bone marrow (73% infiltration), mediastinum, and pleurae were affected.

The prognosis was considered very poor. Treatment had already encompassed FLAG-IDA during the course, and nelarabine monotherapy was initially considered treatment of choice. Led by a brisk CD38 expression on 96% of the cancer cells in the bone marrow, we combined daratumumab, nelarabine, pegylated asparaginase, and dexamethasone as described in Table 1.

Treatment was well tolerated initially, and clinically the patient improved considerably within a few days. A complete remission with a negative bone marrow test after 3 weeks and a negative PET/CT scan after 6 weeks was seen. At this point, Grade I sensory poly-neuropathic symptoms in the hands were reported by the patient (CTCAE v5.0).

SCT was scheduled, and treatment was continued as described in Table 1 for 13 weeks. After nine weeks, the patient had an uncomplicated episode of neutropenic fever.

General weakness interfering with daily life and Grade II sensory polyneuropathy in hands and feet were seen until conditioning consisting of involved-field radiotherapy (mediastinum, 18 Gy in total), TBI (12 Gy in total) (testes 4 Gy), and cyclophosphamide (120 mg/kg).

During conditioning, over a few days, the patient developed a progressive severe neurological disability with a combination of spinal and peripheral ataxia, sensory loss, and pareses of upper and lower extremities. Sphincter functions were impaired. The symptoms were progressive till 3 weeks after SCT and then became stable for months. Some improvement has taken place later, but the patient remains considerably disabled—most neurological symptoms are still Grade III. He is alive with no signs of relapse 3½ years after transplantation. A moderate chronic GvHD is still treated with immunosuppressive drugs.

The neurological impairment was characterized by neurologists after MR scan showing medullopathy, as well as clinical and neurophysiological examinations. It was presumably caused by a combination of nelarabine and radiotherapy as reported earlier [10, 11]. A possible complicating role of daratumumab cannot be excluded but is not considered likely, given the well-known neurotoxic potential of nelarabine.

## 3. Case 2

A 25-year-old female with no medical history presented with T-ALL with 80% bone marrow involvement but no CNS affection. She was treated as per NOPHO2008 protocol. Due to a poor bone marrow response on day 15, she was referred to the high-risk arm aiming for a myeloablative SCT. She received 5 high-risk blocks but remained MRD positive. At SCT, her MRD was 1.7%, but the procedure was carried out, since we lacked an obvious better alternative. Conditioning regimen was etoposide 1800 mg/m^2^ and TBI 12.5 Gy. No GvHD was seen in the course.

After 3 months, she had a bone marrow relapse (35% infiltration on flowcytometry, and 71% of cancer cells were strongly positive for CD38) without CNS infiltration.

As a consequence of the neurotoxicity seen in Case 1, we modified the combination regimen and treated with weekly daratumumab, only two three-day courses of nelarabine, and less dexamethasone as shown in Table 1.

The regimen was well tolerated. After 6 weeks, she had a complete remission, and for the first time in the entire treatment period, a negative MRD (flow cytometry and TCR gene rearrangement PCR) was seen after 9 weeks.

Daratumumab was continued as monotherapy weekly for 8 courses and then every two weeks. After a few weeks, she developed Grade III acute GvHD affecting liver and intestine. Daratumumab was stopped for 2 weeks while GvHD was diagnosed but was then started again. High-dose prednisolone and ciclosporine were administered without response. Four courses of infliximab 10 mg/kg weekly was added, and steroid was tapered. She had two episodes of septicaemia with *Aeromonas* and *Bacteroides*. Furthermore, a fungal infection was suspected and treated with posaconazole. The bone marrow function was weak, but neutrophils responded well to filgrastim, and gradually her condition improved.

We initially aimed for a second SCT, but due to the severe GvHD and her general condition after this complication, we went for long-term daratumumab treatment instead. Unfortunately, the leukemia relapsed 196 days after starting salvage therapy. MRD was 0.047% in multicolour flowcytometry and similar in PCR. At this second relapse, she was physically quite well, and ciclosporine was tapered without recurrence of GvHD. She did not want further medical treatment at this point but received homeopathic therapy abroad.

Ten weeks later, she was admitted with a clinical superior vena cava syndrome. A CT scan revealed large mediastinal lymphomas, pleural effusion, and lymphoma masses in mammary glands, around the large vessels, and within the posterior thoracic wall. She was treated with dexamethasone, nelarabine, and palliative radiotherapy (12 Gy in total). A short relief was seen, but ultimately, she died 5 months after positive MRD was identified.

## 4. Discussion

The two cases suggest a possible role for daratumumab/nelarabine combination in treatment of relapsing T lymphoblastic leukemia/lymphoma as a path to SCT in Case 1. Re-transplant had to be abandoned in Case 2 due to GvHD and the patient's general condition. Nevertheless, the duration of her second remission was long enough for a re-transplant, had it been possible. The usual short duration of CR2, hampering SCT, is a major challenge in these patients, and therefore we consider the apparent effect of combining daratumumab and nelarabine meaningful. In Case 2, however, a possible graft-versus-leukemia effect may have had a role in maintaining the CR2 for several months.

The treatment was effective as re-induction and initially well tolerated even in these heavily treated patients. Neurological side effects seen in Case 1 were most likely caused by an additive effect of nelarabine, total burden of other chemotherapy, and radiotherapy. For this reason, Case 2 only had two courses of nelarabine, and she only had a Grade I sensory polyneuropathy.

There were no indications of interactions between the two drugs, but this topic still needs attention.

A few case reports have suggested that daratumumab monotherapy may be effective as re-induction and palliative therapy in T lymphoblastic leukemia/lymphoma [5–7]. Use of daratumumab/nelarabine combination is addressed in two publications, but in the 4 cases described, the drugs were separated in time, as daratumumab was administered 3 weeks after de-bulking chemotherapy. The results were promising [8, 9]. One case had failed monotherapy with daratumumab just prior to chemotherapy with a large amount of leukemia in the bone marrow, but the drug seemed effective in diminishing MRD afterwards [8].

Conclusively, the role of daratumumab, alone or in combination, in relapsing T lymphoblastic leukemia/lymphoma is not clear at present. A prospective phase 2 trial of daratumumab monotherapy in relapsing ALL has enrolled 47 patients so far and will hopefully bring important new knowledge on monotherapy with daratumumab (ClinicalTrial.gov (NCT03384654)). Combining daratumumab with nelarabine or other chemotherapy may also be an emerging therapeutic option in relapsing T lymphoblastic leukemia/lymphoma, and results so far merit further tests.

## Figures and Tables

**Table 1:** Overview of salvage therapy with daratumumab/nelarabine combination.

	Case 1	Case 2
Daratumumab	16 mg/kg i.v., d1 weekly for 13 weeks (till start of conditioning)	16 mg/kg i.v., d1 weekly for 8 weeks and thereafter every 2 weeks till relapse (total 17 infusions)
Nelarabine	1500 mg/m2, d1, 3, 5 every 3 weeks, 4 courses	1500 mg/m2, d1, 3, 5 every 3 weeks, 2 courses
Dexamethasone	40 mg, d1–4 every 3 weeks, 4 courses	40 mg, d1–4 every two weeks, 2 courses
PEG-asparaginase	1000 mg/m2 i.m., d1 every 3 weeks, 4 courses	not used
PEG-filgrastim	6 mg s.c., d7 every 3 weeks, 4 courses	6 mg s.c., d7 every 3 weeks, 2 courses


Effect on relapse of T-lymphoblast disease	CR : bone marrow after 3 weeks CR : PET/CT scan after 6 weeks	Partial remission after 3 weeks CR, neg. PCR after 6 weeks CR, neg. PCR and FC after 9 weeks Second relapse d196


Side effects, first 3 weeks	Bone marrow impairment, need for transfusions	Fatigue, sore muscles first few days Thrombocytopenia
Side effects, subsequent weeks	Grade I-II sensory polyneuropathy (hands, feet) Bone marrow impairment Uncomplicated neutropenic fever Weakness	Grade III GVHD (liver, intestine) Bacteremia x 2 Bone marrow impairment Grade I sensory polyneuropathy (hands)
Side effects, after conditioning	Grade III-IV neurological impairment (see text)	No re-SCT
